# Prevalence and VP1 Gene Evaluation Analysis of Porcine Sapelovirus in Yunnan Province, China, from 2024 to 2025

**DOI:** 10.3390/v17101336

**Published:** 2025-09-30

**Authors:** Zhanhong Li, Xuyu Tang, Zhenxing Zhang, Pei Zhu, Zhuoran Li, Peng Liu, Qi Yang, Li Meng, Xiutao Sun, Zhen Yang, Qiuyan Yang, Yifang Zhang, Jianling Song

**Affiliations:** 1 Yunnan Tropical and Subtropical Animal Virus Diseases Laboratory, Yunnan Animal Science and Veterinary Institute, Kunming 650224, China; dy081lzh@163.com (Z.L.); zhenxing978@163.com (Z.Z.); zpcau@sina.com (P.Z.); lizhuoran85@126.com (Z.L.); 2 Key Laboratory of Transboundary Animal Diseases Prevention and Control (Co-Construction by Ministry and Province), Kunming 650224, China; 3 School of Animal Medicine, Yunnan Agricultural University, Kunming 650201, China; 4 Mile Preventive and Control Center for Animal Diseases, Mile 652300, China; 5 Honghe Preventive and Control Center for Animal Diseases, Mengzi 661100, China

**Keywords:** porcine sapelovirus, Yunnan province, epidemiology, *VP1* gene, evaluation analysis

## Abstract

Porcine Sapelovirus (PSV) is widely prevalent in pig herds throughout the world and induces diarrhea, encephalomyelitis, respiratory tract symptoms, and reproductive disorders. However, the epidemiological and genetic evolution characteristics of PSV remain unclear in Yunnan Province. In this study, 1622 fecal samples were collected from pig farms in Yunnan Province. PSV and its co-infection rates with other pathogens were detected; then, the PSV *VP1* gene was amplified and sequenced; and the genetic evolution characteristics of the *VP1* gene were analyzed. The overall infection rate of PSV in Yunnan Province was 36.50%, and the differences among regions were significant (*p* < 0.05). The positive rates among different seasons were significantly different (*p* < 0.01), ranging from 73.33% (autumn) to 19.00% (summer). The PSV positive rate in diarrhea samples (47.26%) was significantly higher (*p* < 0.001) than that of non-diarrhea samples (31.77%). The co-infection rates of PSV with porcine rotavirus (PoRV) and PSV with porcine epidemic diarrhea virus (PEDV) were 5.07% and 3.04%. A total of 36 *VP1* sequences were obtained, and the average identity among the 36 sequences was 85.3%, which was higher than that with other reference strains. Phylogenetic analysis revealed that all 36 PSV strains belonged to the PSV-1 genotype. The *VP1* gene was under strong negative selection pressure (average dN/dS = 0.0838); however, the 95th amino acid was under positive selection pressure. Our study revealed the epidemiological, co-infection, and genetic evolution characteristics of PSV in pig herds of Yunnan Province, providing more data for preventing and controlling diarrhea pathogens in pigs.

## 1. Introduction

Porcine Sapelovirus (PSV) is a member of the genus *Sapelovirus* within the family *Picornaviridae* and was previously known as porcine enterovirus 8 (PEV-8) [1]. PSV is a non-enveloped virus comprising a single-strand positive RNA genome of approximately 7.5 kb, and the genome contains a single open reading frame (ORF) flanked by a 5′ and 3′ untranslated region (UTR) with a poly (A) tail [2,3]. The large ORF encodes a polyprotein that proteolytically matures to generate four structural proteins (VP1, VP2, VP3, and VP4) and seven nonstructural proteins (2A to 2C and 3A to 3D). The PSV capsid protein VP1 is located on the surface of the virion and is the most immunodominant protein, containing several major antigenic epitopes. Variation in antigenic epitopes may directly affect the recognition efficiency of the host immune system, and thus, VP1 is a vital target for serological diagnosis and genotyping [4,5]. Based on phylogenetic and genetic analyses of the polyprotein, *P1* and *VP1* genes of PSV sequences, two genotypes (PSV-1 and PSV-2) have been identified [6]. Currently, PSV-1 is widely prevalent worldwide, while PSV-2 has only been detected and reported in Hungary [7].

Domestic pigs and wild boars are natural hosts of PSV [8]. The correlation between PSV prevalence and the age of the animal is still ambiguous; Ákos Boros [7], Sunaga F [9], and Yang T [10] reported a higher prevalence in younger animals (suckling and nursery pigs) compared to fattening pigs, while GY B [11] reported a higher prevalence in growing and finishing pigs. The most common transmission method of PSV is the fecal–oral route [12], while transmission through aerosols and direct contact and vertical transmission are alternative methods [13,14]. PSV infection is associated with various symptoms, ranging from asymptomatic to clinical diseases such as diarrhea, encephalomyelitis, skin lesions, respiratory tract symptoms, and reproductive disorders [12]. Co-infection with other enteric pathogens is always detected [13], making pathogenicity more complex. The first strain of PSV (V13) was isolated from the feces of pigs in the UK [14], and since then, PSVs have been identified in different countries worldwide, with prevalence ranging between 7.1% (India) and 75.5% (Zambia) [3,15]. Epidemiological investigations of PSV have been conducted in many provinces in China, with prevalences of 17.2%, 18.21%, 20.4%, 42.21%, and 61.25% identified in East China, South China, Sichuan, Hunan, and Ningxia province, respectively [16,17].

At present, the scale of the pig-breeding industry in Yunnan Province is expanding continuously, and it has become one of the largest pig-raising provinces in China. However, the epidemiological characteristics of PSV, its genetic evolution patterns, and the details of co-infection with other pathogens remain unclear. In this study, we collected 1622 fecal samples from pig farms in 10 prefectures or counties of Yunnan Province from April 2024 to March 2025. The infection rate of PSV was determined and co-infection with porcine epidemic diarrhea virus (PEDV), transmissible gastroenteritis virus (TGEV), and porcine rotavirus (PoRV) was identified by reverse transcription polymerase chain reaction (RT-PCR). The PSV *VP1* gene was then amplified for sequencing to analyze its genetic evolution characteristics. This study highlights the spatial distribution characteristics, epidemic trends, and characteristics of multi-pathogen co-infection and genetic evolution of PSV in Yunnan Province for the first time.

## 2. Materials and Methods

### 2.1. Sample Collection

From April 2024 to March 2025, 1622 pig fecal samples were collected from 91 pig farms from 10 prefectures in Yunnan Province, among which 957 samples were collected from Mile County, and 218 diarrhea samples and 447 non-diarrhea samples were collected from other areas (Table 1). The samples were transported to the laboratory on dry ice in 5-milliliter centrifuge tubes, and then, they were temporarily stored in a −80 °C refrigerator until extraction and detection of viral nucleic acid.

### 2.2. RNA Extraction and RT-PCR Amplification

One gram of each fecal sample was placed into a 10 mL centrifuge tube with 5 mL 1× PBS buffer, vortexed for 2 min, and then centrifuged at 12,000 r/min for 5 min at 4 °C. Subsequently, the viral RNA was extracted from the supernatant using a MagMAX™-96 Viral RNA Isolation Kit (Applied Biosystem, Foster, CA, USA) following the manual instructions. The extracted RNA was stored at −80 °C until RT-PCR amplification.

PSV nucleic acids in the samples were detected using a PrimeScript^TM^ One step RT-PCR Kit (Takara, Dalian, China) with the primers “pev-8g and pev-8h” (Table 2) reported by Krumbholz [18]. The RT-PCR protocol consisted of an initial reverse transcription step at 50 °C for 30 min and then predenaturation at 94 °C for 3 min, followed by 30 cycles of denaturation at 95 °C for 30 s, annealing at 55 °C for 30 s, and extension at 72 °C for 30 s. After the reaction, the amplified PCR products were analyzed through 1.5% agarose gel electrophoresis and visualized on an ultraviolet-light transilluminator (Bio-Rad Lab., Hercules, CA, USA). In addition, the most common diarrhea-related porcine viruses (PEDV, TGEV, PoRV) were screened from PSV-positive samples through one step RT-PCR using primers reported previously [19].

### 2.3. PSV VP1 Gene Amplification and Sequencing

The *VP1* PSV genes from 38 PSV-positive samples from the different pig farms of different regions were amplified using the reported primer (Table 2) [16] through one step RT-PCR. The amplification protocol consisted of an initial reverse transcription step at 50 °C for 30 min, and then predenaturation at 94 °C for 3 min, followed by 30 cycles of denaturation at 95 °C for 30 s, annealing at 55 °C for 30 s, and extension at 72 °C for 1 min 30 s. After the reaction, the amplified PCR products were analyzed through 1.5% agarose gel electrophoresis and visualized on an ultraviolet-light transilluminator (Bio-Rad Lab., USA). Subsequently the expected special PCR products were sent to Qingke Bioengineering Co., Ltd. (Kunming, China),for Sanger sequencing and sequence assembly using SeqMan in the DNASTAR software package (version 7.10, DNASTAR, Madison, WI, USA).

### 2.4. VP1 Sequence Identity and Phylogenetic Analysis

In total, 136 *VP1* gene sequences of PSV from different countries were downloaded from GenBank as a reference and aligned with the *VP1* gene sequences obtained in this study using MAFFT software (Verxion 7.380) [20]. The sequence identity values were calculated using BioEdit (Version 7.1.3.0) [21], and a line graph was plotted in GraphPad Prism 6 (Version 6.02) and heatmaps were plotted using https://www.bioinformatics.com.cn (last accessed on 10 December 2024), an online platform for data analysis and visualization [22]. The Neighbor-Joining (NJ) phylogenetic tree of the *VP1* genes was generated via MEGA v.6.0 [23] using the model ‘Kimura 2-parameter’ with 1000 replicates in the bootstrap test.

### 2.5. Selection Pressure Analysis of the VP1 Gene of PSV

The selection pressure of the *VP1* gene was analyzed through the Datamonkey online analysis software version 2.0 (accessed on 26 March 2025) [24]. Four methods, including mixed effects model of evolution (MEME), fixed-effects likelihood (FEL), single-likelihood ancestor counting (SLAC), and fast unbiased Bayesian approximation (FUBAR), were used to determine the presence of pervasive purifying and diversifying selection. The confidence levels of the FEL, SLAC, and MEME methods were set to *p* value < 0.1, and the posterior probability threshold for the FUBAR method was set to >0.7. The results of the four analysis methods were compared, and the types and intensities of the selective pressure of the PSV *VP1* gene were analyzed. The selective pressure sites were considered under positive or negative selection when detected as such by at least two different methods.

### 2.6. Predictions of the Structures and the B-Cell Epitopes of VP1 Protein of PSV

The hydrophilicity/hydrophobicity of the PSV-*VP1* protein was analyzed using ProtScale online analysis software (https://web.expasy.org/protscale/protscale-ref.html (accessed on 10 July 2025)) [25]; then, the B-cell epitopes of the PSV-*VP1* protein were predicted through B Cell Epitope Prediction Tools (http://tools.iedb.org/main/bcell/ (accessed on 11 May 2025)) to determine whether the amino acid at the positive selection site is located in the antigenic epitope advantage region. Finally, the tertiary structure models of PSV-*VP1* were predicted using SwissModel.10 [26], and positive-selective-pressure amino acid sites were highlighted in the models.

### 2.7. Statistical Analysis

Infection rates with 95% confidence intervals (95% CI) were calculated using SPSS version 27.0 software (SPSS Corporation, Chicago, IL, USA). Pearson Chi-Square Tests were conducted using SPSS version 27.0 to analyze the significant differences in PSV infection rates among regions, fecal status (diarrheal and non-diarrheal) and seasons. For small samples or samples with zero frequency, the Fisher Exact Test was conducted. A *p*-value less than 0.05 was considered statistically significant.

## 3. Results

### 3.1. Positive Rate and Regional Distribution of PSV in Yunnan Province

**Figure 1:**
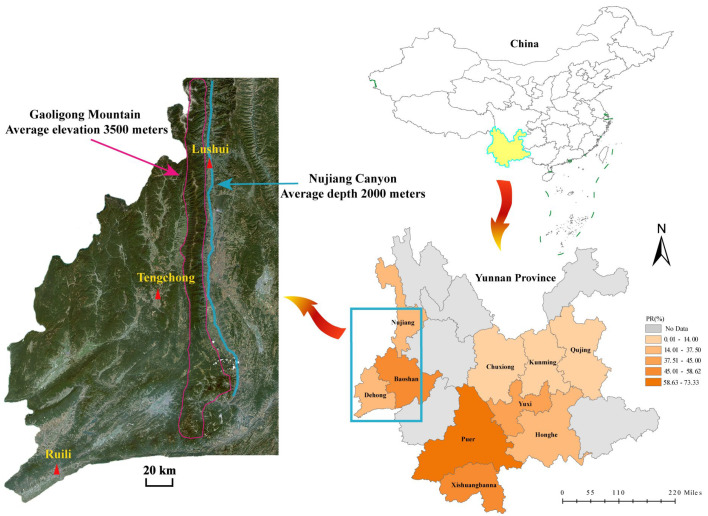
Positivity rate of PSV in pig herds in different regions of Yunnan Province, and the location of the sampling point in Dehong refecture (Ruili county), Baoshan prefecture (Tengchong county), and Nujiang. prefecture (Lushui county).

The overall detection rate of PSV in pig herds of Yunnan Province was 36.50% (592/1622, 95% CI 34.15–38.84), and the positive rates among different regions were significantly different (χ^2^ = 58.842, df = 9, *p* < 0.001). In detail, Puer showed the highest positivity rate of 73.33% (22/30, 95% CI 56.54–90.13), followed by Xishuangbanna at 58.62% (17/29, 95% CI 39.56–77.69). Chuxiong and Kunming showed the lowest positivity rates, both at 10% (3/30, 95% CI 0–21.39). The positive rates in other regions ranged from 14.00% (7/50, 95% CI 4.04–23.96) in Qujing to 57.69% (15/26, 95% CI 37.34–78.04) in Tengchong (Table 3, Figure 1).

The overall positive rate of diarrheal samples was significantly higher than that of non-diarrheal samples (χ^2^ = 14.353, df = 1, *p* < 0.01), at 47.26% (95/201, 95% CI 40.30–54.22) compared to 31.77% (142/447, 95% CI 27.43–36.10) (Table 3). Except for Kunming and Chuxiong, PSV infection was found in both diarrheal and non-diarrheal samples. The positive rate of diarrheal samples in Honghe was significantly higher than that of non-diarrheal samples (*p* < 0.001) (Table 4), while the positive rate of non-diarrheal samples in Nujiang was significantly higher than that of diarrheal samples (*p* < 0.05) (Table 4). In Ruili, Tengchong, Xishuangbanna, Qujing, Yuxi, and Puer, the positive rate of PSV in diarrheal samples was higher than that in non-diarrheal samples, but the difference was not significant (Fisher Exact Test, *p* > 0.05) (Table 4).

**Figure 2:**
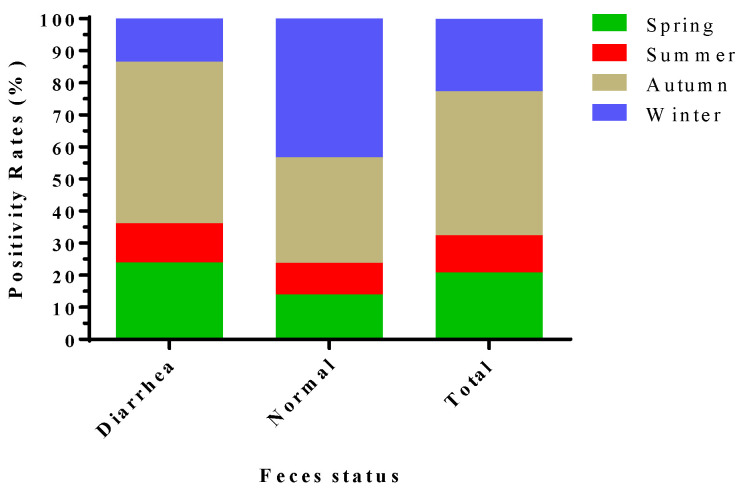
Analysis of the proportion of PSV-positive rates in fecal samples collected from pig herds in Yunnan Province in different seasons from 2024 to 2025.

The positive rates among different seasons were significantly different (χ^2^ = 31.137, df = 3, *p* < 0.01) (Table 1), and are ordered from high to low as follows: 73.33% (22/30, 95% CI 56.54–90.13) (Autumn), 37.06% (531/1433, 95% CI 34.55–39.56) (Winter), 33.90% (20/59, 95% CI 21.46–46.34) (Spring), and 19.00% (19/100, 95% CI 11.18–26.82) (Summer) (Table 3). Further, based on the health status of the fecal samples, the positive rate of the diarrhea samples collected was the highest in autumn (50.92%), while the positive rate of the normal fecal samples collected was the highest in winter (43.24%). In terms of the overall positive rate, that of samples collected in autumn was the highest (44.91%). It is noteworthy that the positive rate of samples collected in summer was the lowest (12.26% for diarrhea and 9.88% for normal) (Figure 2).

### 3.2. Co-Infection Rate with Other Enteric Pathogens

**Figure 3:**
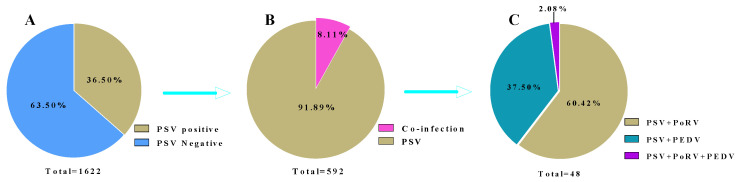
The overall positive rate of PSVand the co-infection ratios of PSV with PEDV, TGEV and PoRV in the fecal samples collected from pig herds of Yunnan Province from 2024 to 2025. (**A**) The overall positive rate of PSV; (**B**) The PSV single-infection and co-infection ratios; (**C**) The co-infection ratios of PSV with PEDV, TGEV and PoRV.

A total of 47 samples from the 592 PSV-nucleic-acid-positive samples exhibited dual infection and 1 sample exhibited triple infection; the total co-infection rate was 8.11% (48/592, 95% CI 6.1–10.68) (Figure 3B). Among the co-infection samples, 29 cases were PSV and PoRV dual infections, accounting for 60.42% (29/48, 95% CI 45.3–73.89) of the total mixed infections, and 18 cases were PSV and PEDV dual infections, accounting for 37.5% (18/48, 95% CI 24.32–52.67) of the total mixed infections. Only 1 sample was triply infected with PSV, PEDV and PoRV, accounting for 2.08% (1/48, 95% CI 0.11–12.48) (Figure 3C). No mixed infections of PSV and TGEV were detected.

### 3.3. VP1 Sequence Identity Analysis

**Figure 4:**
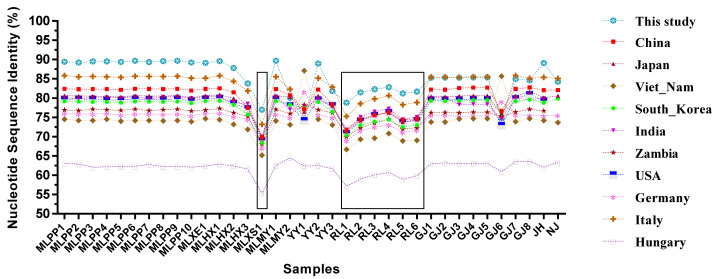
The average nucleotide identity of PSV-*VP1* sequences obtained in this study and that of the reference strains of different countries.

In this study, the *VP1* genes of 38 PSV-positive samples were amplified and sequenced. Except for two samples from Tengchong, all samples were successfully amplified. Ultimately, 36 full-length *VP1* gene sequences were obtained (GenBank accession numbers: PX204884–PX204918). These gene sequences were compared with reference strains from different countries. On average, the sequences obtained in this study showed the highest similarity (85.3%) amongst themselves, while the similarity with *VP1* genes of other reference strains ranged from 61.9% (Hungary strain) to 83.1% (Italy strain) (Table S1, Figure 4). In terms of individual strains, the *VP1* gene sequence of MLXS1 exhibited the highest variability, with its average similarity to the *VP1* genes of other PSV strains ranging from 55.3% (Hungary strain) to 73.2% (Italy strain) (Table S1). Additionally, the six *VP1* sequences (RL1 to RL6) obtained from Ruili also showed relatively low similarity to other PSV strains (Table S1, Figure 4).

**Figure 5:**
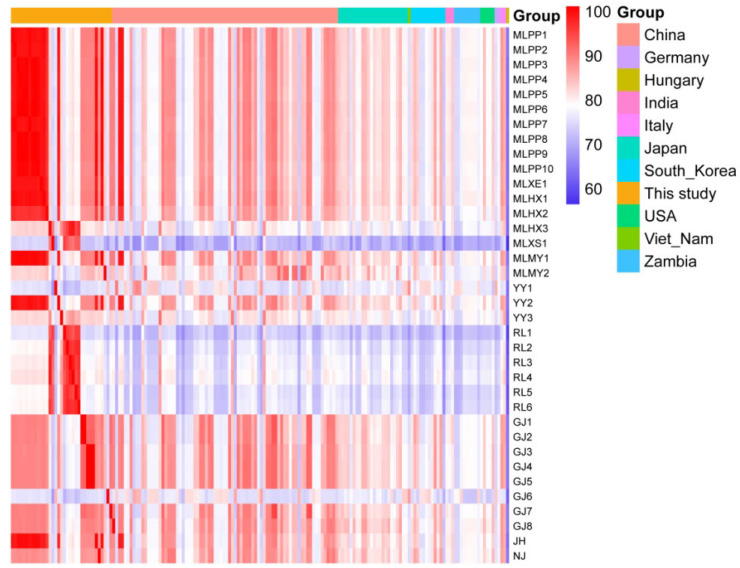
The heat map of the average nucleotide identity of the 36 PSV-*VP1* sequences obtained in this study and that of the reference strain from different countries.

The nucleotide similarity heatmap shows the similarity between the 36 PSV *VP1* gene sequences obtained in this study and the other 136 PSV *VP1* gene sequences from different countries around the world. Except for the 8 sequences obtained from the Ruili-1 to -6, Mile-Xinshao and Gejiu-6 sampling sites, the other 28 PSV *VP1* sequences obtained all showed high similarity with the Chinese strain (Figure 5), while the similarity with *VP1* sequences from other countries was relatively low (Figure 5).

### 3.4. VP1 Gene Phylogenetic Analysis

**Figure 6:**
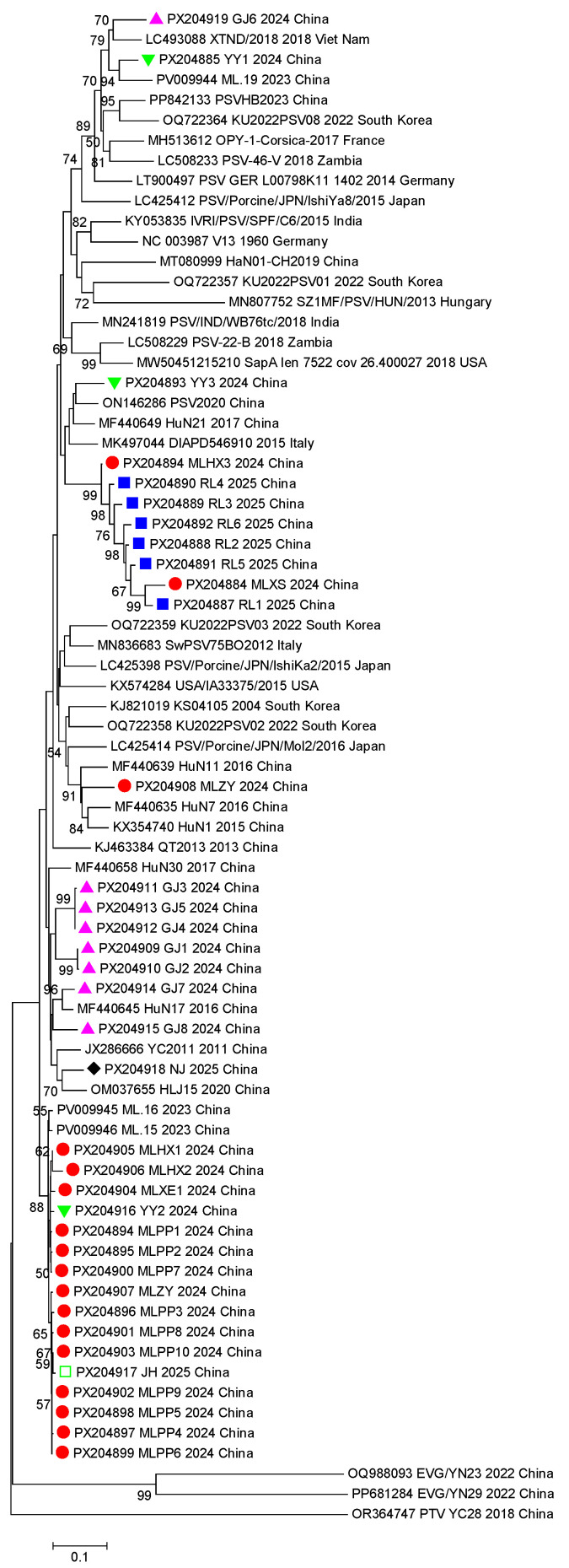
Phylogenetic analysis based on the *VP1* gene sequence of PSV strains detected in the samples and reference strains from GenBank. The phylogenetic tree was constructed by the neighbor-joining method in MEGA 6.0 and tested by bootstrapping 1000 replicates. The sequences obtained from Mile are labeled with a red dot (●), sequences obtained from Gejiu are labeled with a purple regular triangle (▲), sequences obtained from Ruili are labeled with a blue square (■), sequences obtained from Yuanyang are labeled with a green inverted triangle (▼), sequences obtained from Xishuangbanna are labeled with a light-blue five-pointed star (□), sequences obtained from Nujiang are labeled with a black rhombus (◆). The sequences of reference PSV strains are indicated as GenBank accession number, strain name, year, and country. The *VP1* gene of EVG/YN23, EVG/YN29 and PTV/YC28 were used as outgroup to root the phylogenetic tree.

The phylogenetic tree of the PSV *VP1* gene shows that the 36 PSV strains from Yunnan were all located in the PSV-1 branch (Figure 6), indicating that they were all the PSV-1 genotype. PSV strains from different countries were dispersed throughout the phylogenetic tree, suggesting that there is no obvious correlation between the topology of the *VP1* gene and the geographical origin of the strains (Figure 6). Further observation of the distribution of the *VP1* sequences obtained in this study showed that PSV strains from the same sampling location were clustered together in a relatively independent branch (Figure 6). For example, 14 (82.4%) of the 17 *VP1* gene sequences from Mile County were clustered, 5 (71.4%) of the 7 *VP1* gene sequences from Gejiu were clustered, and all 6 *VP1* gene sequences from Ruili were clustered. This seemingly indicates that the dominant PSV strain in a localized region is relatively unitary. It was noted that some of the *VP1* sequences obtained from Haoxi Town (PX204897, MLHX3), XiShao Town (PX204898, MLXS) and Miyang Town (PX204900, MLD2) in Mile County are far away from the sequences obtained from other towns in Mile County, suggesting that there may be more than one epidemic strain of PSV in the pig herds here. A similar situation can be observed the sequences obtained from Yuanyang and Gejiu (Figure 6).

### 3.5. Selection Pressure Analysis of the VP1 Gene

Four methods were used to conduct a selection pressure analysis on the PSV *VP1* protein sequence in Datamonkey online analysis software. A substantial number of negative selection sites were identified via the FUBAR, FEL and SLAC methods, and the total average dN/dS calculated via SLAC was 0.0838, indicating that the PSV *VP1* gene was under strong negative selection pressure. As for positive selection sites, the MEME, FUBAR, FEL and SLAC method identified 24, 1, 0 and 2 sites, respectively (Table 5). The 95th positive selection site was identified via both FUBAR and MEME, while the 86th and 269th sites were only identified by SLAC.

### 3.6. Prediction of the Hydropath, B Cell Epitopes and Tertiary Structure of the PSV VP1 Protein

**Figure 7:**
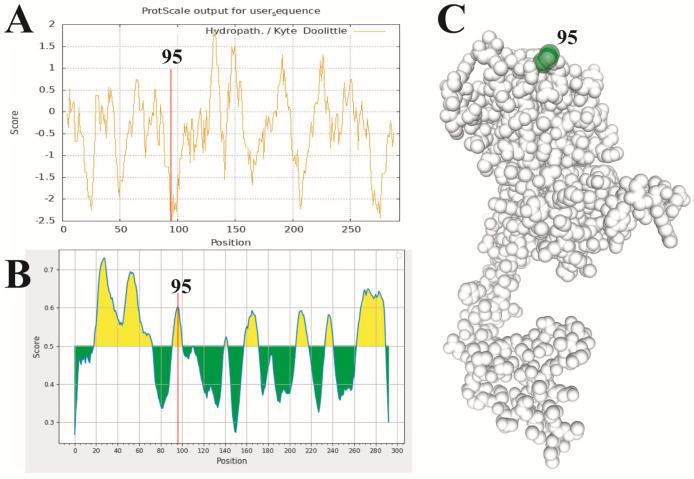
Prediction of the hydropath (**A**), B cell epitopes (**B**) and tertiary structure (**C**) of the VP1 protein of PSV.

Hydropath analysis of the *VP1* protein showed that the average hydropath was −0.4206, indicating that PSV *VP1* is a hydrophilic protein. Notably, the hydropathicity value of the 95th amino acid (−2.467) was the second lowest among all amino acids, only higher than that of the 94th amino acid (−2.50) (Figure 7A). We also predicted the B-cell antigenic epitope-dominant regions of the *VP1* protein of PSV, showing that the 95th amino acid was located in one of the antigenic-epitope-dominant regions (92-YTNPQGQRHL-101) of the PSV *VP1* protein (Figure 7B). Additionally, we used Swiss-model to predict the tertiary structure of the PSV *VP1* protein, showing that the 95th amino acid was located on the surface of the *VP1* protein (Figure 7C). The above results suggest that the 95th amino acid of the *VP1* protein may have been continuously affected by the host immune system during the evolution of PSV, eventually inducing a positive selection mutation.

## 4. Discussion

Since PSV was first discovered in the UK in 1958, it has spread extensively around the world, with infection rates varying among different regions and host populations [27,28,29,30]. In this study, the overall detection rate of PSV in pig herds in Yunnan Province was 36.50% (95% CI 34.15–38.84), higher than that in Shanghai and its surrounding areas (10.47% to 21.58%) [31] and similar to that in Sichuan Province (30.0%) [32]. However, the prevalence of PSV varied significantly among different prefectures (counties) in Yunnan Province: the detection rate was higher in tropical and subtropical border areas such as Puer (73.33%, 95% CI 56.54–90.13) and Xishuangbanna (58.62%, 95% CI 39.56–77.69), while it was lower in plateau areas such as Kunming and Chuxiong (both 10%). We inferred that the possible reason for this difference in geographical distribution is the regional climate. The climates of Puer and Xishuangbanna are more humid than those of Kunming and Chuxiong, and wet climates are more beneficial for virus transmission. The difference in positivity rates between seasons was also significant (*p* < 0.01), with positivity rates in cold seasons (autumn, winter, and spring) higher than those in the warm season (summer). Although there is no direct evidence of this, we speculate that the positivity rates may be correlated with temperature because virus viability is prolonged in low-temperature conditions; however, virus activity is significantly reduced at higher temperatures.

At present, the correlation between PSV infection and porcine diarrhea was still controversial. The most common cases were the co-infection of PSV with other diarrhea pathgens, such as PEDV, TGEV, and PoRV. To determine whether PSV infection is related to porcine diarrhea, we compare the positive rates of PSV single infection in diarrheal and non-diarrheal samples. The results indicated that the positive rates of PSV single infection in diarrheal samples were 47.26% (95% CI 40.30–54.22) and that were 31.77% (95% CI 27.43–36.10%) in non-diarrheal samples, and nine of ten sampling sites showed a higher infection risk for pig herds exhibiting diarrhea symptoms. The positive rate of PSV in diarrheal samples was significantly higher (chi-square test, *p* < 0.001) than that in non-diarrheal samples. According to the results of our study, PSV infection may be associated with porcine diarrhea, which is consistent with the results of Zhang B et al. [33,34]. Nevertheless, just the co-infection pathogens PEDV, TGEV, PRoV were tested, and other enteric pathogens, such as porcine teschoviruses (PTV), porcine sapovirus (PSaV), porcine astrovirus (PoAstV), and porcine deltacoronavirus (PDCoV) were excluded. Therefore, there may be other pathogens beyond “PEDV, TGEV, and PRoV” co-infected in the diarrhea samples. Moreover, the PSV positive rate was as high as 31.77% (95% CI 27.43–36.10%) in the non-diarrheal samples, and the extreme case, the positive rate in the non-diarrhea samples from Nujiang was 60.00% (95% CI 36.48–83.52). Generally, the relationship between PSV infection and porcine diarrhea is not absolute. It may be affected by various factors, such as the difference between pandemic strains, the age of animals and co-infection pathogens.

It is well known that geographical barrier is critical to block the transmission of the pathogens, and the regional evolution of viruses often leads to the formation of geographic branches (topotypes) with significantly different characteristics [35,36]. Based on the results of this study, we speculate that the evolution of the PSV *VP1* gene was also influenced by certain geographical characteristics. Firstly, the average similarity between the six *VP1* sequences obtained from Ruili and the other 30 *VP1* sequences obtained in this study was only 78.37%, and the highest similarity with strains from other regions in China was only 80.2%. In addition, the average nucleotide identity with global reference strains was between 61.9% and 83.1%, much lower than the average identity of 95.8% among the six Ruili strains. Further phylogenetic analysis revealed that the six sequences from Ruili formed a relatively independent branch—this may be related to the endemic prevalence of the Ruili PSV strains. Additionally, only one *VP1* sequence was amplified from the three PSV-positive samples from Nujiang, and this sequence was relatively distant from the other Yunnan strains in the phylogenetic tree, suggesting that the PSV strains prevalent in Nujiang shared strong territorial characteristics. Moreover, amplification failed for both PSV-positive samples from Tengchong. We speculate that this might be due to the mismatch between the primer binding regions of the PSV strains prevalent in Tengchong and the sequences of the primers used for amplification. Geographically, Ruili is located in a relatively remote corner in the southwest of Yunnan Province; both Nujiang and Tengchong are separated from other regions by Gaoligong Mountain (average elevation: 3500 m), the inconvenient transportation may hamper the trade of pigs. Moreover, the dominant pig breeds of these regions are the local breeds, such as Diannan small-ear pig and Gaoligongshan pig, and the frequency of introducing boar from other regions is very low. All the above factors may reduce the frequency of genetic exchange between locally prevalent and external PSV strains, leading to the long-term prevalence of the virus in a relatively closed geographical environment and the gradual accumulation of specific mutations. This ultimately induced the unique genetic variation characteristics of the PSV strains in Ruili, Nujiang and Tengchong.

The evolution of the PSV *VP1* gene might be related to the breeding scale. Samples from Mile and Gejiu were mainly collected from medium- and large-scale pig farms. A total of 14 of the 17 (82.4%) *VP1* gene sequences obtained from Mile clustered into one group, while 5 of the 7 sequences (71.4%) obtained from Gejiu clustered together; this implies that a single dominant strain might prevail in the local area. A possible reason for this might be the rapid spread of the virus due to concentrated breeding. In sharp contrast, samples from Yuanyang were collected from three small-scale or scattered farms, and the three *VP1* sequences were located on distant branches of the phylogenetic tree, highlighting the genetic diversity of PSV in Yuanyang. This geographical evolutionary difference is significant for epidemiological surveillance: for regions with genetic homogeneity, the epidemic risk of the pathogens can be assessed through monitoring the variation in the dominant strain, while in regions with a high genetic diversity, high-throughput sequencing technology may be more suitable to monitor the dynamics of the virus population.

The selection pressure of the PSV *VP1* gene was mainly attributed to purifying selection pressure. This indicates that although the variation in the PSV *VP1* nucleotide and amino acid sequence was much greater than that of other genes, amino acid mutations mainly occurred through synonymous substitution. We speculate that this may be because the immune pressure on naturally prevalent PSV is relatively low. Currently, no vaccines are used for PSV worldwide, so the immune pressure only comes from the natural immune system of the host and is not affected by artificial immune pressure. However, we found that the 95th amino acid was under positive selection pressure. Hydrophilicity analysis shows that the hydrophilicity index of the amino acid at this position is very low, only −2.467, indicating that this position indeed has high hydrophilicity and thus that it may have good immunogenicity. Through antigenic epitope prediction, the 95th amino acid was found to be located in a potential antigenic epitope region. It was revealed through the predicted tertiary structure of the *VP1* protein that the 95th amino acid is located on the surface of this protein. These results suggest that the 95th amino acid may be directly affected by the host immune system and is under strong host immune pressure. Previous studies have shown that amino acid variations in the main immunogenic proteins of viruses caused by host immune pressure may change the charge, hydrophobicity or spatial conformation of the amino acids, causing them to evade recognition by the host immune system [37] or causing a reduction in the binding efficiency of neutralizing antibodies [38], eventually leading to immune escape. However, the effects of the mutation of the 95th amino acid in the PSV *VP1* protein need to be verified through further experiments such as site-directed mutagenesis.

## 5. Conclusions

PSV is widely prevalent (overall positivity rate: 36.50%) in pig herds in Yunnan Province, and the differences in positivity rate among different regions, seasons or feces statuses (diarrhea and non-diarrhea) were all significant. Co-infection rates were 5.07% (PSV with PoRV) and 3.04% (PSV with PEDV). A total of 36 *VP1* sequences analyzed in this study showed high homology, all belonging to the PSV-1 genotype, and they are under strong negative selection pressure, except the 95th amino acid, which is under positive selection pressure.

## Figures and Tables

**Table 1:** Information of feces samples collected in this researh.

Prefecture	County	Town	Number of Pig Farm	Number of Sample	Numer of Pigs
Honghe	Mile	Pengpu, Xinshao, Hongxi, Zhuyuan, Xiyi, Xier, Xisan, Dongshan, Jiangbian, Wushan, Xunjiansi, Dongfeng	44	957	1500–8000
	Geji	Datun	30	276	500–6000
	Yuanyang	Nansha, Ganiang	7	82	50–200
Dehong	Ruili	Mengmao	5	52	/
Boshan	Tengchong	Beihai	1	26	/
Xishuangbannan	Jinghong	Jinghong	1	29	/
Nujiang	Lushui	Laowo	1	40	/
Kunming	Xundian	Jingsuo	1	30	/
Qujing	Zhanyi	Zhanyi	1	50	/
Yuxi	Jiangchuan	Jiangchuan	1	20	/
Chuxiong	Lufeng	Jingshan	1	30	/
Puer	Simao	Simao	1	30	/
Total	/	/	91	1622	/

**Table 2:** Primers used in this researh.

Primer Name	Sequence (5′-3′)	Product Size (bp)	Purpose	Reference
pev-8g	ATGGCAGTAGCGTGGCGAGCTAT	212	Screening PSV infection	[18]
pev-8h	GTAATGCCAAGAGCATGCGCCA			
*VP1*-F	ATTGCCTAYACACCACCTGG	1324	Amplificating *VP1* gene	[16]
*VP1*-R	GCAGGTCTTCTCCCACAAAC			

**Table 3:** Comparison of PSV detection rates among regions, fecal status and seasons.

Variables	Category ^a^	No. Tested ^b^	No. Positive	Positivity Rate (%) (95% CI)	Heterogeneity (χ^2^/df/*p*)
Regions	Honghe	1315	484	36.81 (34.20–39.42)	58.842/9/<0.01
	Dehong	52	17	32.69 (19.51–45.88)	
	Boshan	26	15	57.69 (37.34–78.04)	
	Xishuangbannan	29	17	58.62 (39.56–77.69)	
	Nujiang	40	15	37.50 (21.82–53.18)	
	Kunming	30	3	10.00 (0.00–21.39)	
	Qujing	50	7	14.00 (4.04–23.96)	
	Yuxi	20	9	45.00 (21.11–68.89)	
	Chuxiong	30	3	10.00 (0.00–21.39)	
	Puer	30	22	73.33 (56.54–90.13)	
Fecal status ^c^	Diarrhea	201	95	47.26 (40.30–54.22)	14.353/1/<0.01
	Normal	447	142	31.77 (27.43–36.10)	
Season	Spring	59	20	33.90 (21.46–46.34)	31.137/3/<0.01
	Summer	100	19	19.00 (11.18–26.82)	
	Autumn	30	22	73.33 (56.54–90.13)	
	Winter	1433	531	37.06 (34.55–39.56)	
Total	/	1622	592	36.50 (34.15–38.84)	/

^a^ Total 1315 samples were collected from Honghe prefecture, and the samples were collected from three counties (Mile, n = 957; Gejiu, n = 276, and Yuanyang, n = 82), respectively. ^b^ The fecal samples of Mile county were randomly collected, and the status of the samples was indeterminate. Therefore, they were not used to analyze the correlation between PSV infection and diarrhea. ^c^ The mix-infection fecal samples were excluded.

**Table 4:** Positivity rate of PSV in the diarrhea or normal feces collected from different regions of Yunnan Province.

Region	Month	Season	Fecal Status	No. Tested	No. Positive	Positivity Rate (%) (95% CI)	Heterogeneity (χ^2^/df/*p*)
Honghe	2024-12	Winter	Diarrhea	52	43	82.69 (72.05–93.33)	44.247/1/<0.001
			Normal	306	103	33.66 (28.34–38.99)	
Dehong	2025-1	Winter	Diarrhea	32	13	40.63 (22.64–58.62)	2.379/1/0.123
			Normal	20	4	20.00 (7.93–39.21)	
Boshan	2025-1	Winter	Diarrhea	15	9	60.00 (31.92–88.08)	0.077/1/0.781
			Normal	11	6	54.55 (19.46–89.63)	
Xishuang bannan	2025-3	Spring	Diarrhea	19	12	63.16 (39.27–87.05)	0.468/1/0.494
			Normal	10	5	50.00 (12.30–87.70)	
Nujiang	2025-1	Winter	Diarrhea	20	3	15.00 (0–32.15)	8.640/1/0.03
			Normal	20	12	60.00 (36.48–83.52)	
Kunming	2024-4	Spring	Diarrhea	20	3	15.00 (0–32.15)	1.667/1/0.197
			Normal	10	0	0.00 (/)	
Qujing	2024-7	Summer	Diarrhea	15	4	26.67 (1.32–52.02)	2.856/1/0.091
			Normal	35	3	8.57 (0–18.33)	
Yuxi	2024-7	Summer	Diarrhea	10	6	60.00 (23.06–96.94)	1.184/1/0.178
			Normal	10	3	30.00 (4.56–64.56)	
Chuxiong	2024-7	Summer	Diarrhea	15	3	20.00 (0–42.93)	3.333/1/0.068
			Normal	15	0	0.00 (/)	
Puer	2024-9	Autumn	Diarrhea	20	16	80.00 (60.79–99.21)	1.364/1/0.243
			Normal	10	6	60.00 (23.06–96.94)	

**Table 5:** Positive selection sites in the PSV *VP1* gene.

Position aa	Methods							
	FUBAR		FEL		SLAC		MEME	
	dN/dS	Post. Pr	dN/dS	*p*-Value	dN/dS	*p*-Value	ω+	*p*-Value
10	-	-	-	-	-	-	9.597	0.008
14	-	-	-	-	-	-	279.998	0.001
24	-	-	-	-	-	-	16.504	0.095
28	-	-	-	-	-	-	5.260	0.028
29	-	-	-	-	-	-	340.648	0.001
43	-	-	-	-	-	-	15.655	0.005
66	-	-	-	-	-	-	205.843	0.007
77	-	-	-	-	-	-	106.758	0.002
86	-	-	-	-	4.948	0.067	-	-
89	-	-	-	-	-	-	124.021	0
95	3.578	0.988	-	-	-	-	11.175	0
98	-	-	-	-	-	-	9.287	0.038
102	-	-	-	-	-	-	27.608	0.004
113	-	-	-	-	-	-	16.391	0.019
128	-	-	-	-	-	-	260.906	0.039
133	-	-	-	-	-	-	22.093	0.093
164	-	-	-	-	-	-	27.084	0.003
173	-	-	-	-	-	-	30.708	0.014
184	-	-	-	-	-	-	42.496	0.023
213	-	-	-	-	-	-	10.195	0.032
228	-	-	-	-	-	-	33.849	0
238	-	-	-	-	-	-	9.121	0.052
269	-	-	-	-	Infinite	0.095	-	-
290	-	-	-	-	-	-	122.086	0.004
291	-	-	-	-	-	-	36.941	0.042
292	-	-	-	-	-	-	26.788	0.007

aa: amino acid; dN/dS = ω+: Rate of nonsynonymous mutation/Synonymous mutation; Post. Pr: posterior probability.

## Data Availability

The original contributions presented in this study are included in the article. Further inquiries can be directed to the corresponding author.

## Supplementary Materials

The following supporting information can be downloaded at https://www.mdpi.com/article/10.3390/v17101336/s1, Table S1: Nucleotide sequence identity (%) between PSV VP1 genes obtained from this study and reference PSV strains.
