# Supplementary material for: Prevalence and VP1 Gene Evaluation Analysis of Porcine Sapelovirus in Yunnan Province, China, from 2024 to 2025
Source: Viruses. 2025 Sep 30;17(10):1336. doi: 10.3390/v17101336 (PMC12567820; doi:10.3390/v17101336)
Supplement: Supplementary file 1 [file viruses-17-01336-s001.zip › viruses-3862730-supplementary.pdf]

Table S1. Nucleotide sequence identity (%) between PSV VP1 genes obtained from this study and reference PSV strains.

Samples	The nucleotide sequence identity (%)										
(Gene Bank No.)	This study	China	Japan	Viet_Nam	South_Korea	India	Zambia	USA	Germany	Italy	Hungary
MLPP1	89.4	82.5	80.6	74.5	79.2	80.1	76.9	79.7	76.1	85.9	63.0
MLPP2	89.2	82.3	80.6	74.2	79.1	79.9	76.8	79.8	75.9	85.5	62.9
MLPP3	89.5	82.3	80.5	74.2	79.0	80.3	77.1	79.7	75.9	85.6	62.1
MLPP4	89.5	82.4	80.5	74.5	79.0	80.2	77.0	79.5	76.0	85.5	62.2
MLPP5	89.4	82.1	80.3	74.0	78.8	80.1	76.8	79.5	75.6	85.4	62.3
MLPP6	89.7	82.5	80.6	74.2	79.2	80.4	77.1	79.8	75.9	85.7	62.3
MLPP7	89.3	82.4	80.6	74.2	79.1	79.9	76.8	79.7	75.9	85.6	62.8
MLPP8	89.6	82.3	80.5	74.1	79.0	80.3	77.0	79.6	75.8	85.6	62.2
MLPP9	89.7	82.5	80.6	74.2	79.2	80.4	77.1	79.8	75.9	85.7	62.3
MLPP10	89.2	82.0	80.1	73.9	78.7	80.0	76.6	79.3	75.4	85.2	62.1
MLXE1	89.2	82.4	80.6	74.7	79.2	80.4	76.9	79.8	76.1	85.2	62.4
MLHX1	89.6	82.6	80.9	74.4	79.3	80.4	77.3	80.0	76.1	85.8	62.9
MLHX2	87.8	81.5	79.9	73.2	78.3	78.8	76.2	78.7	75.2	84.4	62.4

MLHX3	83.8	77.6	77.5	71.9	75.8	78.6	75.3	77.3	74.5	81.9	61.6
MLXS	77.0	70.0	69.6	65.2	68.4	70.1	68.0	69.0	67.0	73.2	55.3
MLD1	89.7	82.4	80.6	74.1	79.2	80.4	77.0	79.8	75.8	85.6	62.5
MLD2	80.1	80.7	80.3	73.1	78.3	77.1	76.0	78.1	74.9	82.3	64.5
YY1	74.6	77.2	76.5	87.1	77.4	77.6	78.3	74.2	81.7	74.8	62.4
YY2	89.0	82.2	80.6	74.5	79.0	79.7	77.0	79.6	75.7	85.2	62.5
YY3	81.8	78.4	78.1	73.0	76.5	77.8	76.2	78.1	74.9	82.9	61.7
RL1	78.8	71.8	71.4	66.7	70.4	71.7	70.0	70.9	69.0	75.3	57.2
RL2	81.5	74.5	74.2	69.3	72.9	75.1	72.3	74.1	71.6	78.6	59.0
RL3	82.3	75.6	75.6	69.6	73.9	76.5	73.5	75.5	72.5	79.8	60.1
RL4	82.9	76.4	76.2	70.8	74.5	77.3	74.6	76.3	73.1	80.7	60.7
RL5	81.2	74.3	73.8	68.9	72.5	74.5	72.0	73.7	71.1	78.3	58.9
RL6	81.7	74.7	74.4	69.0	73.0	75.1	72.3	74.2	71.7	78.9	59.9
GJ1	85.3	82.2	80.4	73.8	79.4	80.1	76.2	79.5	75.6	85.5	63.0
GJ2	85.3	82.2	80.4	73.8	79.3	80.1	76.3	79.5	75.6	85.5	63.2
GJ3	85.3	82.6	80.6	74.6	79.4	78.3	76.2	79.6	75.3	85.4	63.0

GJ4	85.4	82.7	80.7	74.7	79.5	78.4	76.3	79.7	75.4	85.5	63.1
GJ5	85.4	82.7	80.7	74.7	79.5	78.4	76.3	79.7	75.4	85.5	63.1
GJ51	74.7	76.7	76.1	85.7	76.0	74.6	75.3	73.0	79.1	75.1	60.9
GJ52	85.0	82.4	80.6	73.9	79.1	80.1	76.4	79.9	75.7	85.9	63.6
GJ53	84.6	82.8	81.6	74.7	79.6	79.5	77.1	80.9	75.5	85.2	63.6
JH	89.1	82.1	80.1	74.2	78.8	80.0	76.7	79.3	75.4	85.4	62.1
NJ	84.3	82.1	80.6	73.7	79.9	79.6	76.7	80.1	75.6	85.1	63.4
Average	85.3	80.1	78.8	73.6	77.5	78.4	75.7	78.0	74.9	83.1	61.9
